# Periplocymarin Plays an Efficacious Cardiotonic Role *via* Promoting Calcium Influx

**DOI:** 10.3389/fphar.2020.01292

**Published:** 2020-08-19

**Authors:** Weijing Yun, Lei Qian, Yanyan Cheng, Weiwei Tao, Ruqiang Yuan, Hu Xu

**Affiliations:** ^1^Advanced Institute for Medical Sciences, Dalian Medical University, Dalian, China; ^2^College of Nursing, Dalian Medical University, Dalian, China

**Keywords:** periplocymarin, cardiac function, Ca^2+^ influx, cardiomyocyte, blood pressure

## Abstract

Periplocymarin, which belongs to cardiac glycosides, is an effective component extracted from Periplocae Cortex. However, its cardiovascular effects remain unidentified. In the present study, injection of periplocymarin (5 mg/kg) through external jugular vein immediately increased the mean arterial pressure (MAP) in anesthetized C57BL/6 mice. *Ex vivo* experiments using mouse mesenteric artery rings were conducted to validate the role of periplocymarin on blood vessels. However, periplocymarin failed to induce vasoconstriction directly, and had no effects on vasoconstriction induced by phenylephrine (Phe) and angiotensin II (Ang II). In addition, vasodilatation induced by acetylcholine (Ach) was insusceptible to periplocymarin. Echocardiography was used to evaluate the effects of periplocymarin on cardiac function. The results showed that the injection of periplocymarin significantly increase the ejection fraction (EF) in mice without changing the heart rate. *In vitro* studies using isolated neonatal rat ventricular myocytes (NRVMs) revealed that periplocymarin transiently increased the intracellular Ca^2+^ concentration observed by confocal microscope. But in Ca^2+^-free buffer, this phenomenon vanished. Besides, inhibition of sodium potassium-activated adenosine triphosphatase (Na^+^-K^+^-ATPase) by digoxin significantly suppressed the increase of MAP and EF in mice, and the influx of Ca^2+^ in cardiomyocytes, mediated by periplocymarin. Collectively, these findings demonstrated that periplocymarin increased the contractility of myocardium by promoting the Ca^2+^ influx of cardiomyocytes *via* targeting on Na^+^-K^+^-ATPase, which indirectly led to the instantaneous rise of blood pressure.

## Introduction

Cardiovascular diseases (CVDs) are the most common causes of death worldwide. CVDs are disorders of the heart and blood vessels, including heart failure (HF), hypertension, and other conditions (Tzoulaki et al., 2016; Zhao et al., 2019). HF is the culmination of CVDs, characterized by complex clinical syndromes, such as dyspnea, orthopnea, lower limb swelling, elevated jugular venous pressure, and pulmonary congestion (Tanai and Frantz, 2015; Ponikowski et al., 2016). Besides ACEI (angiotensin-converting enzyme inhibitor), ARB (angiotensin receptor blocker), beta-blocker, and MRA (mineralocorticoid receptor antagonist), positive inotropic drugs are also used in the treatment of HF under certain circumstances.

Cardiac glycosides, including digoxin and ouabain, exhibit high selectivity to heart and exert a positive inotropic action by enhancing myocardial contractility and thus improve cardiac pumping function (Alogna et al., 2016). Hence, they convey vitally important effects as therapeutic agents for the treatment of HF. Mechanically, cardiac glycosides effectively inhibit sodium potassium-activated adenosine triphosphatase (Na^+^-K^+^-ATPase), resulting in disrupting the active transport of Na^+^ from the intracellular to the extracellular space and altering the balance between intracellular Na^+^ and K^+^. The increase of intracellular Na^+^ concentration and the decrease of intracellular K^+^ concentration lead to an increase of intracellular Ca^2+^ concentration by reducing Ca^2+^ efflux *via* Na^+^-Ca^2+^ exchange system (Ambrosy et al., 2014; Stucky and Goldberger, 2015). Accordingly, digoxin was widely used as an inhibitor of Na^+^-K^+^-ATPase in studies (Ashbrook et al., 2016). Accumulating evidence shows that the precise roles and mechanisms of each cardiac glycoside are distinctive in cardiovascular system. For example, endogenous cardiotonic ouabain can alter intracellular calcium (Ca^2+^) homeostasis in smooth muscle cells, and thus affect the constriction of vessels, eventually resulting in approximately 30% increase in systolic blood pressure in rodent model after 1 week of ouabain treatment (Pulgar et al., 2013; Zulian et al., 2013). However, digoxin has no effect on cytolic free Ca^2+^ concentration in vascular smooth muscle cells and does not alter blood pressure (Zulian et al., 2013).

Unfortunately, despite the beneficial features of traditional positive inotropic drugs, including digitalis, phosphodiesterase inhibitors, and catecholamines, they also exhibit adverse effects (Alogna et al., 2016). It has been proposed that some cardiac glycosides increase oxygen consumption and calcium load of the heart. These potential risks greatly restricted the clinical application of these drugs, which can only be used as short-term therapeutic measures. Therefore, there is an urgent need to develop new positive inotropic drugs with low toxicity and a wide safety range.

Periplocae Cortex is an important ingredient of some heart-strengthening formulas, which is widely used in the treatment of cardiac dysfunction in traditional Chinese medicine (Wang et al., 2017). However, the effects of its active compounds have not been fully identified or reported. Periplocymarin, which possesses the basic structure of cardiac glucoside, is isolated from Periplocae Cortex (Itokawa et al., 1988), while the physiological/pathophysiological roles of periplocymarin *in vivo* are largely unknown. Previous studies demonstrated that periplocymarin exerted a potent anti-cancer activity by inhibiting proliferation and inducing apoptosis of tumor cells (Bloise et al., 2009; Martey et al., 2014). Regrettably, although the pharmacokinetics has been investigated by Yan et al. in animals, the cardiovascular role of periplocymarin as a cardiac glucoside, especially on HF or acute heart failure (AHF), remains to be clarified (Yan et al., 2016).

In this study, we demonstrated that periplocymarin increased myocardial contractility *via* enhancing Ca^2+^ influx by inhibiting Na^+^-K^+^-ATPase in cardiomyocytes. Our research provided a novel insight for exploring better inotropic drugs.

## Methods and Materials

### Chemicals and Drugs

Periplocymarin, phenylephrine, and acetylcholine were purchased from Sigma-Aldrich. The purity of all compounds was more than 98% determined by HPLC analysis. Digoxin, KB-R7943 were purchased from MedChemExpress.

### Animals

Adult (8–10 weeks) male C57BL/6 mice were maintained at the Animal Center of Dalian Medical University. Animals were housed at an ambient temperature of 23°C with 12-h light/dark cycles and given a fresh diet and sterile water every other day. The animal protocols were approved by the Animal Care and Use Review Committee of Dalian Medical University. All procedures followed the Guide for the Care and Use of Laboratory Animals (National Institutes of Health).

### Isolation and Culture of Primary NRVMs

Primary NRVMs were cultured as previously described (Nyui et al., 1997). Briefly, the hearts of 1–3-day-old neonatal Sprague-Dawley rats were digested with trypsin and collagenase. The dissociated cells were plated on 100-mm culture dishes in Dulbecco’s modified eagle medium (DMEM) with 10% fetal bovine serum (FBS) for 3 h in a humidified incubator (37°C, 5% CO_2_). The nonattached cardiomyocyte-rich fraction was plated on plastic dishes with DMEM containing 10% FBS and 1% penicillin-streptomycin, and BrdU (100 μM) was added during the first 48 h to reduce the non-myocytes. The culture medium was changed 48 h.

### Internal Carotid Blood Pressure Measurements

Male C57BL/6J mice aged 8 weeks and with 22 to 25 g body weight were used. Mice were anesthetized by intraperitoneal injecting of 70 mg/kg pentobarbital and placed on a temperature-controlled pad. A jugular vein catheter was placed for the infusion of test periplocymarin. PE-10 tubing was inserted into the right carotid artery, and blood pressure was measured using a transducer connected to a blood pressure analyzer (BL-420N, TECHMAN) (Zhang et al., 2000). Mean arterial pressure (MAP) was recorded for 10 to 30 min until stable values were obtained, and then 40 μl periplocymarin (5 mg/kg) or digoxin were injected *via* the jugular vein.

### Mesenteric Arterial Vascular Reactivity

Mice were killed by CO_2_ inhalation, and the vessels were rapidly harvested and cleaned from adherent connective and fat tissue. Mesenteric arteries were cut into rings (2 mm in length) quickly in cold Krebs solution. Each ring was suspended between two tungsten wires in chambers of a Multi Myograph System (610 M, Danish Myo Technology A/S, Aarhus N, Denmark) for the measurement of isometric force. Each chamber was filled with 5 ml of Krebs bicarbonate buffer and maintained at 37°C. During the entire experiment, the buffer was continuously oxygenated with 95% O_2_ plus 5% CO_2_ gas (Wang et al., 2015). The vasoconstriction effect of periplocymarin was performed after the vessels were well balanced. The gradient of periplocymarin was added every 2 min. To examine the effect of periplocymarin on the vasodilatory effect of acetylcholine (Ach) or contractile response to phenylephrine (Phe) and Angiotensin II (Ang II), the arteries were incubated with periplocymarin (10 μM) for 30 min before treatments.

### Echocardiography

Echocardiography was performed to evaluate cardiac function after periplocymarin or digoxin administration (Vevo 3100, VisualSonics Inc., Toronto, Canada). In brief, mice were anesthetized with pentobarbital (70 mg/kg) and a jugular vein catheter was placed for infusion of drugs. Parasternal short-axis images were acquired in B-mode with appropriate positioning of the scan head and the maximum left ventricular (LV) length identified. In this view, the M-mode cursor was positioned perpendicular to the maximum LV dimension in end-diastole and systole, and M-mode images were obtained for measuring wall thickness and chamber dimensions before and after periplocymarin or digoxin treatment. The images were measured using Vevo 3100 software (version 1.5.0).

### Measurement of Ca2+ in Cardiomyocytes by Fluorescence Microscopy

Intracellular Ca^2+^ in cardiomyocytes was measured as described previously (Cheang et al., 2013). Cultured cardiomyocytes seeded on the confocal dish were incubated with 2.5 μM Fluo-4 AM in DMEM/HG at 37°C for 1 h. The excess indicator was then washed with Tyrode’s solution containing 5 mM Ca^2+^ or Ca^2+^-free Tyrode’s solution and incubated for 20 min. For digoxin or KB-R7943 pretreatment, cardiomyocytes were incubated by Tyrode’s solution (5 mM Ca^2+^) containing digoxin (10 μM) or KB-R7943 (10 μM) for 20 min after wash. Then the fluorescence images were obtained using an inverted confocal microscope (SP8, Leica, Germany) with a 40× oil immersion objective at 90 frames/min with the excitation laser at 488 nm and 505–530 nm emission. Ca^2+^ influx was stimulated by the addition of 10 µM periplocymarin or digoxin. Changes in Ca^2+^ was expressed as a ratio (F1/F0) by comparing the fluorescence before (F0) and after (F1) adding periplocymarin.

### Molecular Docking

The rigid docking analysis was performed by AutoDock 4.2 with MGL tools 1.5.6 (The Scripps Research Institutes, San Diego, CA, USA). The PDB files of periplocymarin and digoxin were produced using Chem3D Pro software. The crystal structure of Sodium/potassium-transporting ATPase subunit alpha-1 (PDB-code 4ret) was downloaded from RCSB Protein Data Bank. Following the requirement of docking study, ions, water molecules, and non-standard amino acid residues were removed from the proteins. For the docking case, the model with the lowest energy was selected as the binding mode for analysis. The output from AutoDock was rendered with the PyMol program.

### Statistical Analysis

Quantitative data are expressed as the mean ± SEM. Student’s t-test was used to analyze the statistical significance of the differences between the two groups. The dose-response curves were analyzed using two-way ANOVA. P < 0.05 was considered significant. Nonquantitative results are representative of at least three independent experiments.

## Results

### The Mean Arterial Pressure (MAP) Was Increased by Intravenous Administration of Periplocymarin

**Figure 1:**
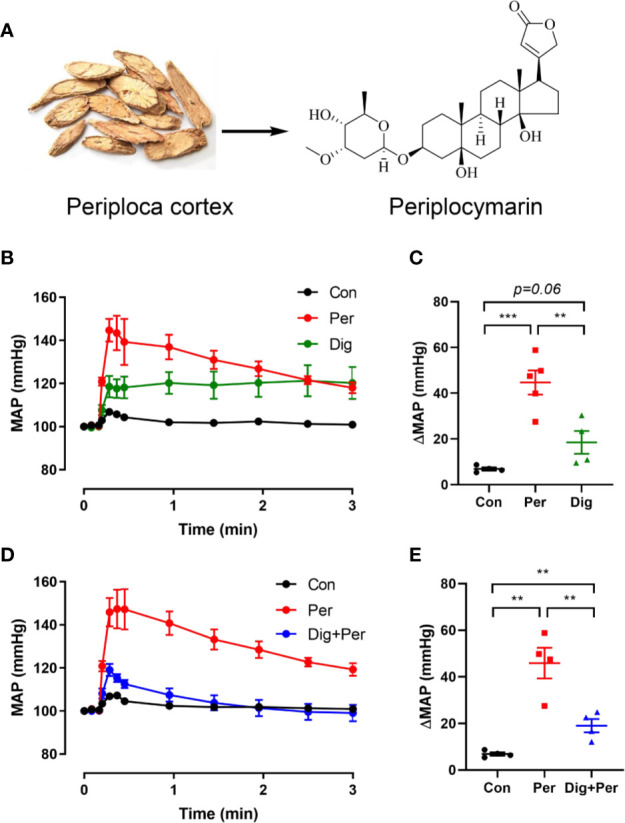
The structure of periplocymarin and its effect on the mean arterial pressure (MAP). **(A)**. The structure of periplocymarin. **(B)**. Control vehicle (10% DMSO), periplocymarin (5 mg/kg, in 10% DMSO) or digoxin (5 mg/kg, in 10% DMSO) was administered by intravenous injection. Mean arterial pressure (MAP) was monitored by intracarotid catheterization before and during intravenous injection. **(C)**. Changes of MAP after treatment with periplocymarin or digoxin. Data shown are means ± SEM; n = 4, **p < 0.01, ***p < 0.001. **(D)**. Effects of periplocymarin on mean arterial pressure (MAP) after digoxin pretreatment for 10 min. Data shown are means ± SEM; n = 4, ***p < 0.001. **(E)**. Changes of MAP after administration in different treatment groups. Data shown are means ± SEM; n = 4, **p < 0.01.

As shown in **Figure 1A**, periplocymarin was extracted from Periplocae Cortex and shares the basic structure of cardiac glucoside. To confirm whether periplocymarin affects hemodynamics, after intravenous administration of periplocymarin (5 mg/kg) or digoxin (5 mg/kg) in anesthetized mice, the MAP was measured. Compared with the digoxin group, elevation of the MAP was more prominent in response to periplocymarin (**Figures 1B, C**). Additionally, pretreatment with digoxin for 10 min significantly suppressed the elevation of MAP induced by periplocymarin (**Figures 1D, E**). Since digoxin inhibited Na^+^-K^+^-ATPase, this data indicated that periplocymarin may also target on Na^+^-K^+^-ATPase. In summary, these results suggested that periplocymarin exerted a pressor effect.

### Periplocymarin Did Not Cause the Contraction of Mesenteric Artery

**Figure 2:**
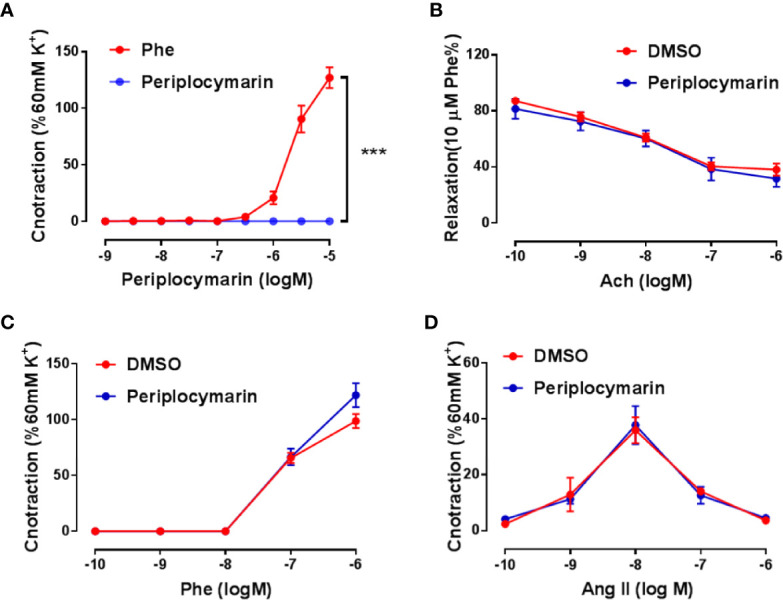
Vascular reactivity was unaffected by periplocymarin treatment. **(A)**. The mesenteric artery rings were treated by the cumulative addition of phenylephrine (Phe) or periplocymarin. Data shown are means ± SEM; n = 4, ***p < 0.001 *vs.* Phe. **(B)**. The mesenteric artery rings were pre-pretreated with vehicle (0.1% DMSO) or periplocymarin (10 µM) for 30 min and contracted by Phe (10 μM), when sustained contraction was obtained, cumulative addition of acetylcholine (Ach) was performed. Data shown are means ± SEM; n = 4. **(C, D)**. Reactivity of endothelium-intact rings treated with vehicle (DMSO) or periplocymarin, followed by the cumulative addition of Phe **(C)** and Ang II **(D)**. Data shown are means ± SEM; n = 4.

Enhanced vasoconstriction of the resistance artery is a critical factor accounting for blood pressure increase. To determine whether periplocymarin affects vascular tension, we performed *ex vivo* experiments using mouse mesenteric arteries. Compared to Phe, which induced remarkable vasoconstriction, periplocymarin failed to induce vasoconstriction (**Figure 2A**). Next, we checked whether periplocymarin affected vasodilation induced by Ach. However, pretreatment with periplocymarin has no effect on Ach-induced mesenteric arteries dilation (**Figure 2B**). Furthermore, periplocymarin pre-incubation had no effect on the vasoconstriction induced by Phe or Ang II (**Figures 2C, D**). These data suggested that the elevation of blood pressure caused by periplocymarin in mice was not associated with vasoconstriction of the resistance artery.

### Periplocymarin Enhanced Myocardium Contractive

**Figure 3:**
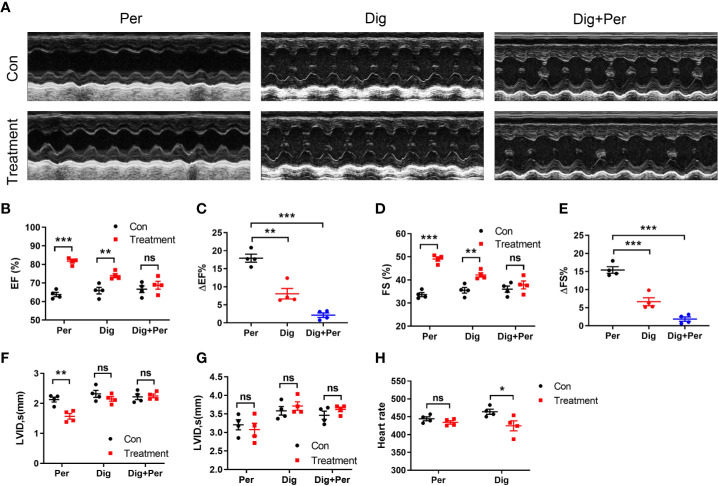
Echocardiography was performed before and after administration of periplocymarin and digoxin (5 mg/kg). **(A)**. Representative echocardiographic photos in M-mode. **(B)**. Ejection fraction (EF%). Data shown are means ± SEM; n = 4, **p < 0.01, ***p < 0.001 *vs.* control. **(C)**. Changes of ejection fraction before and after administration in different treatment groups. Data shown are means ± SEM; n = 4, **p < 0.01, ***p < 0.001 *vs.* periplocymarin. **(D)**. Fractional shortening (FS%). Data shown are means ± SEM; n = 4, **p < 0.01, ***p < 0.001 *vs.* control. **(E)**. Changes of fractional shortening before and after administration in different treatment groups. Data shown are means ± SEM; n = 4, ***p < 0.001*vs.* periplocymarin. **(F)**. Left ventricular end-systolic internal diameter (LVID; s). Data shown are means ± SEM; n = 4, **p < 0.01 *vs.* control. **(G)**. Left ventricular end-diastolic internal diameter (LVID; d). Data shown are means ± SEM; n = 4. **(H)**. Effects of periplocymarin and digoxin on heart rate. Data shown are means ± SEM; n = 4, *p < 0.05 *vs.* control. ns, no significance.

Cardiac output is another dominant factor in the regulation of blood pressure. To evaluate the effect of periplocymarin on cardiac function, we continuously performed echocardiography in the anesthetized mice before and after periplocymarin administration. Compared with the digoxin group, which served as a positive control (**Figure 3A**), the EF was more significantly promoted after administration of periplocymarin (**Figures 3B, C**), as well as the FS (**Figures 3D, E**). However, the effect of periplocymarin on cardiac contractility was remarkably attenuated after digoxin pretreatment, revealed by net changes of EF and FS (**Figures 3C, E**). Meanwhile, the left ventricular end-systolic internal diameter (LVID, s) was obviously shortened after periplocymarin administration (**Figure 3F**), while the left ventricular end-diastolic internal diameter (LVID, d) remained unchanged (**Figure 3G**). This phenomenon was not observed in the digoxin-treated group. In addition, the administration of digoxin significantly reduced the heart rate, while there was no obvious effect of periplocymarin on heart rate (**Figure 3H**). In summary, the systolic function was enhanced but the diastolic function remained unchanged after periplocymarin administration, which suggested that periplocymarin exerted a cardiotonic role.

### Periplocymarin Induced Calcium Influx in NRVMs

**Figure 4:**
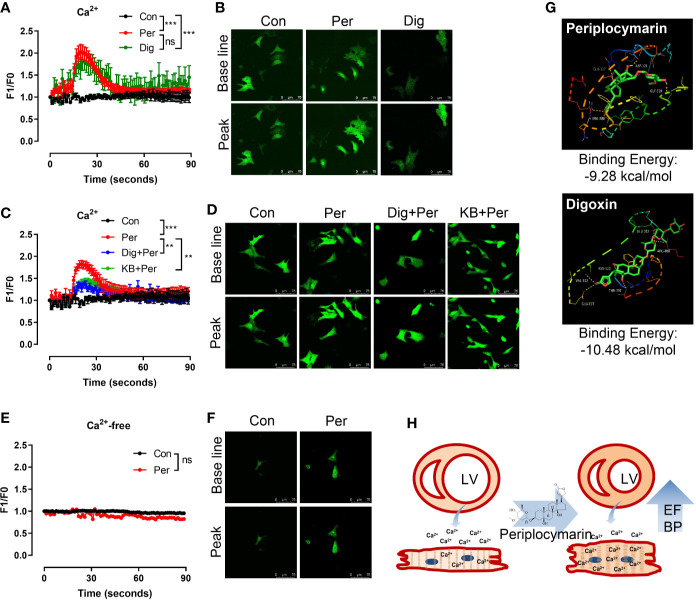
Periplocymarin promoted Ca^2+^ influx in cardiomyocytes. **(A, B)**. Fluo-4 AM fluorescence intensity showing Ca^2+^ concentration changes in cardiomyocytes the addition of periplocymarin (10 μM) or digoxin (10 μM) in Ca^2+^ containing Tyrode’s solution (A). Representative fluorescence images of baseline and the peak points were shown **(B)**. Data shown are means ± SEM; n = 3–6, ***p < 0.001. **(C, D)**. Fluo-4 AM fluorescence intensity showing that the ability of periplocymarin to promote calcium influx was weakened after digoxin or KB-R7943 pretreatment **(C)**. Representative fluorescence images of baseline and the peak points were shown **(D)**. Data shown are means ± SEM; n = 3–6, **p < 0.01, ***p < 0.001. **(E, F)**. Fluo-4 AM fluorescence intensity showing Ca^2+^ concentration changes in cardiomyocytes the addition of periplocymarin (10 μM) in Ca^2+^-free Tyrode’s solution **(E)**. Representative fluorescence images of before and after periplocymarin treatment were shown **(F)**. Data shown are means ± SEM; n = 3–6. **(G)**. The schematic and the three-dimensional diagram of the hydrogen bond interaction between ligands (Periplocymarin, Digoxin) and Na^+^-K^+^-ATPase subunit alpha-1 Chains A. **(H)**. Graphic illustration of the effect of periplocymarin on heart. Periplocymarin increases the concentration of Ca^2+^ in cardiomyocytes, resulting in the contraction of cardiac muscle and increasing the ejection fraction of heart, and thus increasing blood pressure. LV, left ventricle; EF, ejection fraction; BP, blood pressure.

The increase of intracellular Ca^2+^ is a primary and essential process for myocardial contraction. In order to explore the cardiotonic mechanism of periplocymarin, we examined the effect of periplocymarin on intracellular Ca^2+^ levels in isolated NRVMs. The results showed that both digoxin and periplocymarin leaded comparable increases of intracellular Ca^2+^ of NRVMs in Tyrode’s solution containing 5 mM Ca^2+^ (**Figures 4A, B**). The results showed that digoxin, as an inhibitor of Na^+^-K^+^-ATPase, could partly antagonize the effect of periplocymarin. At the same time, KB-R7943 (an inhibitor of Na^+^-Ca^2+^ exchanger) could sufficiently decrease the Ca^2+^ influx mediated by periplocymarin in cardiomyocytes (**Figures 4C, D**). However, the intracellular Ca^2+^ concentration of NRVMs did not change when they were treated by periplocymarin in Ca^2+^-free Tyrode’s solution (**Figures 4E, F**). These results indicated that periplocymarin promoted Ca^2+^ influx in NRVMs.

Based on the above results, we speculated that digoxin and periplocymarin had the same target in cardiomyocytes, so we calculated the binding ability of periplocymarin towards Na^+^-K^+^-ATPase. As shown in **Figure 4G**, the binding energy of digoxin and periplocymarin towards Na^+^-K^+^-ATPase subunit alpha-1 was −10.48 kcal/mol, and −9.28 kcal/mol respectively by molecular docking. Periplocymarin formed four hydrogen bonds with the Na^+^-K^+^-ATPase subunit alpha-1 Chains A (GLN117, ASP121, GLY319, and ARG880). According to the docking results, periplocymarin had strong hydrogen bonding and hydrophobic effects, despite the fact that periplocymarin has slightly lower binding energy towards Na^+^-K^+^-ATPase compared with digoxin. This result further suggested that Na^+^-K^+^-ATPase was the target of periplocymarin.

## Discussion and Conclusion

Periplocae Cortex has a long history as traditional Chinese medicine (TCM) for the treatment of autoimmune diseases (Guo et al., 2013). It was first recorded in *Shen Nong Ben Cao Jing* 2,000 years ago. At present, more than 100 compounds have been identified from Periplocae Cortex, among which some C21-steroidal glycosides and terpenoids have specific immunosuppressive activity (Feng et al., 2008; Guo et al., 2013). In addition to its anti-rheumatic effect, Periplocae Cortex has been shown to possess promising effect on improving cardiac function (He et al., 2015). However, with most of the experiments using a crude extract of Periplocae Cortex, we hardly identify which compound in Periplocae Cortex improves cardiac function. In this study, we employed periplocymarin, a monomer component extracted from Periplocae Cortex. Currently, there are few studies on periplocymarin, most of which focus on its antitumor effects, while its role in cardiovascular disease remains largely unknown (Shiferaw et al., 2003; Zhang et al., 2016). The present study showed that acute administration of periplocymarin facilitated the cardiac function, resulting in an increase of MAP in mice. The underlying mechanism is that periplocymarin increases Ca^2+^ influx *via*, at least partially, Na^+^-K^+^-ATPase in cardiomyocytes.

It has been well documented that peripheral vasoconstriction could lead to an increase of blood pressure (Johnson et al., 1974; Nemes et al., 1980; Nguyen Dinh Cat et al., 2010). Upon the intravenous injection of periplocymarin, a dramatical elevation of MAP was observed in mice. To validate whether periplocymarin could enhance vasoconstriction, we performed experiments on the mesenteric artery ring. Unfortunately, periplocymarin failed to mediate vasoconstriction compared with Phe. In addition, neither vasoconstrictors (Ang II and Phe)-mediated vasoconstriction nor vasodilator (Ach)-induced vasodilation was affected by periplocymarin, which was consistent with previous findings that digoxin failed to stimulate constriction in mesenteric arteries (Sjogren et al., 2016). However, additional investigations are required to assess the long-term effect of periplocymarin on vasoconstriction and vasodilation.

According to published investigations, heart rate and cardiac output are critical factors that directly affect blood pressure (Davis and Sundt, 1980; Thorvaldson et al., 1989; Magder, 2016). In order to evaluate whether cardiac function of mice is affected by periplocymarin, echocardiography was performed and analysis revealed that intravenous administration of periplocymarin had no obvious effect on heart rate, while EF was significantly increased due to the increase of FS. Compared with periplocymarin, digoxin treatment led to a less increase of EF and FS, and a significant decrease of heart rate. This result indicated that periplocymarin appears to serve as a more promising powerful cardiotonic.

It is well known that the increase of myocardial contractility is mainly due to the increase of Ca^2+^ in cardiomyocytes (Chung et al., 2016; Rougier and Abriel, 2016). The main factors of the increase of Ca^2+^ in cardiomyocytes are Ca^2+^ influx and release of intracellular sarcoplasmic reticulum. In order to investigate the effect of periplocymarin on intracellular Ca^2+^ concentration in cardiomyocytes, confocal microscope was employed to evaluate intracellular Ca^2+^ levels in NRVMs. We found that in the presence of Ca^2+^, periplocymarin could increase the intracellular Ca^2+^ in NRVMs, while the cytolic Ca^2+^ was unchanged in Ca^2+^-free buffer, which suggested that periplocymarin could increase the concentration of Ca^2+^ in cardiomyocytes by enhancing Ca^2+^ influx, but not by promoting the release of Ca^2+^ in the sarcoplasmic reticulum calcium pool. A previous study reported that periplocymarin inhibited Na^+^-K^+^-ATPase (Bloise et al., 2009). It was consistent with digoxin, which increases the Ca^2+^ concentration of cardiomyocytes by inhibiting the activity of Na^+^-K^+^-ATPase (Ambrosy et al., 2014). In our study, we used digoxin as an inhibitor of Na^+^-K^+^-ATPase (Stucky and Goldberger, 2015), pretreatment of it significantly suppressed periplocymarin-mediated increase of MAP and EF, and Ca^2+^ influx. Additionally, pretreatment with KB-R7943 remarkably inhibited periplocymarin-induced Ca^2+^ influx in cardiomyocytes. The result of AutoDock showed that periplocymarin had excellent binding energy (−9.28 kcal/mol) with Na^+^-K^+^-ATPase, which was comparable to the digoxin (−10.48 kcal/mol). These results further supported the point that periplocymarin was also an inhibitor of Na^+^-K^+^-ATPase.

Both digoxin and periplocymarin exert positive inotropic effects on the heart, but their metabolic characteristics *in vivo* are distinct. Due to the half-life of digoxin is about 36 h, periplocymarin is only 2 h, there is an advantage that it can be rapidly metabolized after playing a role as cardiotonic, and thus avoid the occurrence of toxic side effects (Hanratty et al., 2000; Yan et al., 2016). P-glycoprotein (P-gp) and drug-metabolizing CYP450s work in tandem to reduce drug bioavailability (Benet, 2009). However, when the principal CYP isoforms (CYP1A2, CYP2C9, CYP2C19, CYP2D6, and CYP3A4) are inhibited, the drug clearance is reduced, leading to an accumulation of drug in the plasma, which can result in adverse drug effects or toxicities (Desta et al., 2001; Huang et al., 2007). According to previous studies, periplocymarin does not inhibit CYP1A2, CYP2C9, CYP2C19, CYP2D6, and CYP3A4 during intestinal absorption (Martey et al., 2014). Therefore, periplocymarin might be a new-found safe and effective cardiotonic drug, which deserves more attention.

Digoxin was used to treat heart failure and supraventricular tachyarrhythmias for a long time. However, it has been proposed that the clinical use of digoxin has been limited by the unique pharmacokinetic properties, electrolyte-dependent effects, P-gp drug interactions, and narrow therapeutic index (Scalese and Salvatore, 2017). Although digoxin has been used for a long time, new cardiotonic drugs with fewer adverse effects are urgently in need, for the treatment of heart failure.

Taken together, we proved that periplocymarin was an effective ingredient for cardiotonic action of Periplocae Cortex. The cardiotonic mechanism of periplocymarin was to increase the concentration of Ca^2+^ in cardiomyocytes by targeting on Na^+^-K^+^-ATPase. This led to the increase of myocardial contractility, resulting in the enhancement of EF of heart, and thus elevated blood pressure (**Figure 4H**). Our findings highlighted the potential value of periplocymarin for treatment of cardiac insufficiency or cardiogenic hypotension.

## Data Availability Statement

The raw data supporting the conclusions of this article will be made available by the authors, without undue reservation.

## Ethics Statement

The animal study was reviewed and approved by Committee of Dalian Medical University.

## Author Contributions

RY performed the animal experiments. LQ did the mesenteric arterial vascular reactivity assay. YC did the cell experiments. WY analyzed data. WT prepared the materials. HX designed the experiments and analyzed data and wrote the manuscript.

## Funding

This work was supported by the National Natural Science Foundation of China Grants (81900267), Doctoral Scientific Research Initiation Fund of Liaoning Province (2019-BS-078), Scientific Research Fund Project of Liaoning Provincial Department of Education (LZ2019014), Liaoning Natural Science Foundation (20180550642), and the Educational Commission of Liaoning Province, China (LQ2017044).

## Conflict of Interest

The authors declare that the research was conducted in the absence of any commercial or financial relationships that could be construed as a potential conflict of interest.
